# Journal Club: Prehospital Detection of Large Vessel Occlusion Stroke With Electroencephalography: Results of the ELECTRA-STROKE Study

**DOI:** 10.1212/WNL.0000000000209587

**Published:** 2024-06-13

**Authors:** Marieta Peycheva, Andrea Seiler, Franca Wagner, Linxin Li, Mirjam R. Heldner

**Affiliations:** From the Department of Neurology (M.P.), Medical University of Plovdiv; Research Institute at Medical University of Plovdiv (M.P.), Bulgaria; Department of Neurology (A.S., M.R.H.), and Institute of Diagnostic and Interventional Neuroradiology (F.W.), Inselspital, University Hospital and University of Bern, Switzerland; and Wolfson Centre for the Prevention of Stroke and Dementia (L.L.), Nuffield Department of Clinical Neurosciences, Wolfson Building-John Radcliffe Hospital, University of Oxford, United Kingdom.

## Abstract

The ELECTRA-STROKE study investigated the potential of EEG for prehospital triage of patients with ischemic stroke due to large vessel occlusion (LVO), in which fast triage to stroke centers for endovascular treatment is crucial. The study was conducted in 4 phases, and this Journal Club article focuses on the fourth phase in the prehospital setting with suspected stroke patients. An EEG cap with dry electrodes was used to measure brain activity. The main focus was on the diagnostic accuracy of the theta/alpha ratio, which yielded an area under the receiver operator characteristic curve (AUC) of 0.80. Secondary endpoints, particularly the Brain Symmetry Index (a quantified EEG interhemispheric cortical power asymmetry index) in the delta frequency band, showed an AUC of 0.91. Despite the convenient study design and user-friendly EEG device, limitations include a single-arm design, a relatively small sample size, and exclusions due to data quality issues. The usefulness of EEG in the detection of neuronal changes based on brain ischemia was highlighted, but uncertainties remain regarding its use in certain patient groups. The improvements in the Brain Symmetry Index from phase 3 to 4 of the study indicate the potential for further refinement and investigation of combined methods to improve diagnostic accuracy. The study provides insight into the role of EEG in prehospital stroke detection, recognizing both the strengths and limitations. Overall, the study contributes to understanding the promise of EEG in optimizing LVO stroke triage and urges further refinement and exploration of complementary diagnostic approaches.

## Introduction

Endovascular treatment (EVT) is the gold standard for managing strokes due to large vessel occlusion (LVO),^1,2^ but the success of treatment is time-dependent.^3^ Accurate prehospital triage is crucial to ensure rapid recognition of these patients for transferring to thrombectomy-capable centers.^4-7^

This Journal Club article discusses the ELECTRA-STROKE study,^8^ which assessed the diagnostic accuracy and feasibility of EEG as a potential simple prehospital tool for detecting anterior circulation LVO.

## Hypothesis and Study Design

The study hypothesized that an EEG with dry electrodes, which is sensitive to a reduction in cerebral blood flow, might provide a high diagnostic accuracy in the detection of anterior circulation LVO in patients with suspected stroke and could therefore be applied as a prehospital triage tool to optimize the decision for direct transport to a thrombectomy-capable center.

ELECTRA-STROKE is a prospective, multicenter, nonrandomized, single-arm diagnostic Dutch study with blinded outcome assessment.^8-11^ It is based on the concept that EEG is good in depicting neuronal changes caused by brain ischemia.^12^ Previous studies have identified EEG patterns in stroke patients, emphasizing an increase in slow waves (theta, delta) and a decrease in fast waves (alpha, beta).^13^ This supports the use of the theta/alpha ratio as an EEG marker for detecting LVO in the hyperacute phase. Moreover, acute stroke-induced differences in EEG activity may also result in interhemispheric asymmetry, which can be potentially measured by the Brain Symmetry Index (BSI).

In an earlier phase of the study, the theta/alpha ratio was found to be the most accurate EEG feature for diagnosing LVO stroke in the anterior circulation in the emergency department (AUC = 0.83/sensitivity = 75%/specificity = 81%).^9-11^ In this current study, the investigators tested the diagnostic accuracy of the theta/alpha ratio as well as other potential markers in a prehospital setting.

## Methods

### Patient Population

Adult patients with clinically suspected stroke as judged by the paramedics, and in whom the onset of symptoms or the time of last seen well was less than 24 hours prior to the EEG recording, were included. Written informed consent was obtained from all patients or their legal representative.

### Study Procedures

After 1 hour of EEG training prior to the study, the paramedics performed an EEG using an EEG cap with dry electrodes, covering the vascular territory of the middle cerebral artery (FC3/FC4/CP3/CP4/FT7/FT8/TP7/TP8). EEG records the patterns of electrical impulses generated by the neurons through electrodes attached to the scalp. These electrical signals can be categorized into different frequency bands (alpha, beta, theta, and delta waves). All patients were subsequently admitted to the hospital and underwent a CT scan, with/without CT angiography (CTA)/CT perfusion to confirm the final diagnosis.

### Definitions and Outcomes

The primary endpoint was the diagnostic accuracy of dry electrode EEG to distinguish anterior circulation LVO stroke from other strokes and mimics. In addition to the theta/alpha ratio,^9-11^ a range of other markers were also explored, including the relative delta = 1–4 Hz, theta = 4–8 Hz, alpha = 8–13 Hz, beta = 13–18 Hz, normalized delta/alpha ratio, normalized (delta/theta)/(alpha/beta) ratio, and pairwise derived BSI.

An adjudication committee including stroke neurologists and neuroradiologists determined the side of the vessel occlusion blinded to EEG results. Occlusions of the intracranial part of the internal carotid artery, the first or proximal second segment of the middle cerebral artery or the first segment of the anterior cerebral artery were defined as LVO. Patients without CTA due to suspected non-LVO stroke were coded as non-LVO stroke.

### Statistical Analysis

After the interim analysis, the sample size was increased from 222 to 386 patients due to a lower than expected incidence of LVO stroke (5% vs 7%) and a higher than expected dropout rate (55% vs 20%).

To evaluate the diagnostic accuracies of the detected EEG features, the investigators calculated the area under the receiver operator characteristic curve (AUC) for each marker. High sensitivity and specificity were defined as ≥70% and ≥80%, respectively. AUC assesses the overall discriminatory performance of a diagnostic test, plotting true positive rate (sensitivity) against false positive rate (1-specificity). An AUC value closer to 1 indicates better discriminatory power in distinguishing patients with a specific disease.

## Results

Of the 386 patients recruited, 212 were included in the final analysis. The main reason for exclusion was insufficient EEG quality or technical EEG failure (32%). Excluded patients were more often female (59% vs 42%), had an LVO stroke (9% vs 3%), and more often had long hair (32% vs 15%). Of the included patients, 3% had an LVO stroke, 51% a non-LVO stroke, 15% a TIA, 4% a hemorrhagic stroke, and 27% a stroke mimic.

In the primary analysis, the AUC of the theta/alpha ratio was 0.80 (95% CI 0.58–1.00) with a sensitivity of 50% (19%–81%), a specificity of 83% (77%–88%), a positive predictive value of 9% (3%–22%), and a negative predictive value of 98% (95%–99%). Among secondary endpoints, BSI had the highest diagnostic accuracy for LVO stroke detection, with an AUC of 0.91 (95% CI 0.73–1.00), a sensitivity of 80% (38%–96%), and a specificity of 93% (88%–96%).

## Interpretation

The ELECTRA-STROKE study^8^ has enhanced our understanding of the feasibility and potential clinical utility of dry electrode EEG as a prehospital triage tool for identifying LVO in the anterior circulation in patients with suspected stroke.

The main strength of the study is the carefully executed multiphase design, facilitating systematic investigation of different research questions and enabling the selection of reliable EEG markers. Another strength is the specially developed EEG device, which is lightweight, portable, and easily implementable by paramedics after just a 1-hour training session. The cap with dry electrodes has several advantages over wet electrodes as they are quick to apply with better penetration through the hairs.^9^ While the learning curve, which indicates improved performance over time, suggests that further adjustments could potentially increase accuracy, retrospective EEG interpretation by the research team relied on expertise and time. Moving forward, recognition algorithms could automate EEG feature calculations, although their accuracy has not been thoroughly investigated yet.^10^

The study design and participant characteristics should be considered when interpreting the results. First, the single-arm study design without randomization presents challenges in establishing causal relationships between the use of the EEG triaging tool and improved patient outcomes. Second, the participation of only 2 out of 25 ambulance services in the region limits generalizability of findings, although the included patients were largely representative of the expected population with suspected strokes. Third, the sample size is relatively small compared to the low LVO incidence, resulting in a wide 95% CI for key measures. This variability diminishes the precision of estimates and may hinder the ability to draw definitive conclusions regarding the effectiveness of the EEG triaging tool. Fourth, the significant number of patient exclusions, particularly linked to LVO cases, is notable. The low rate of good quality EEG recordings emphasizes the necessity for further optimization of the technology, particularly in women and in patients with long hair. In addition, future studies should assess interrater and intrarater reliability to ensure consistency in interpretation. Fifth, CTA was selectively performed in patients with suspected LVO, potentially leading to misdiagnosis and overestimation of specificity. Sixth, electrodes were exclusively placed over the middle cerebral territory. Future research could investigate if accuracy increases by incorporating electrodes in the anterior and posterior cerebral territories. Finally, the study lacked a comparative analysis between EEG markers and established clinical scoring systems, such as RACE/ACT-FAST^4,5^ or somatosensory evoked potentials,^8,14,15^ nor did it integrate them. Future research should incorporate these elements to provide a more comprehensive understanding of the utility of EEG-based triaging in clinical practice.

The key marker of interest in this study was the theta/alpha ratio, which has also been shown to have moderate diagnostic accuracy for LVO stroke in other studies (AUC = 0.69).^14^ In the ELECTRA-STROKE study, despite overall good performance, the sensitivity of the theta/alpha ratio was only 50%, posing a risk of failing to detect LVO in those with LVO stroke and leading to potential misclassification during patient transfer. The reassuringly high specificity of the theta/alpha ratio in this study suggests a low rate of false positive patients. Improved sensitivity while maintaining high specificity will be crucial for this technique, and exploring combinations of different EEG markers may be beneficial. With regard to appropriate discrimination and referral of LVO strokes to thrombectomy-capable centers, the triage tools should provide accuracy with high values for AUC, sensitivity, and positive predictive value. The authors aimed at a sensitivity of ≥70%, which they failed to achieve in their study.

In the current study, BSI showed promise with an AUC of 0.91. Notably, BSI's performance was suboptimal during an earlier phase (AUC = 0.38), but the authors attribute this improvement to enhanced data quality. Further studies are necessary to explore whether BSI can serve as another useful marker.

Other challenges in using EEG as a screening tool include the reliability in patients with preexisting brain changes due to previous stroke, patients with stroke in the posterior circulation, patients with hemorrhagic stroke, other brain diseases, and trauma.^13^

Additional considerations also arise given the extended intervention window and ongoing trials of EVT in patients with low NIHSS scores. This emphasizes the importance of identifying LVO irrespective of the traditional time window and symptom severity. Unfortunately, the sensitivity of the main EEG marker studied was not ideal for this purpose.

## Appendix Authors

**Table:**

Name	Location	Contribution
Marieta Peycheva, MD, PhD	Department of Neurology, Medical University of Plovdiv, Bulgaria; Research Institute at Medical University of Plovdiv, Bulgaria	Drafting/revising the manuscript, interpretation of data, accepts responsibility for conduct of research, and final approval
Andrea Seiler, MD	Department of Neurology, Inselspital, University Hospital and University of Bern, Switzerland	Drafting/revising the manuscript, interpretation of data, accepts responsibility for conduct of research, and final approval
Franca Wagner, MD	Institute of Diagnostic and Interventional Neuroradiology, Inselspital, University Hospital and University of Bern, Switzerland	Drafting/revising the manuscript, interpretation of data, accepts responsibility for conduct of research, and final approval
Linxin Li, DPhil	Wolfson Centre for the Prevention of Stroke and Dementia, Nuffield Department of Clinical Neurosciences, Wolfson Building-John Radcliffe Hospital, University of Oxford, United Kingdom	Drafting/revising the manuscript, interpretation of data, accepts responsibility for conduct of research, and final approval
Mirjam R. Heldner, MSc, MD	Department of Neurology, Inselspital, University Hospital and University of Bern, Switzerland	Drafting/revising the manuscript, interpretation of data, accepts responsibility for conduct of research, and final approval
